# Atrial wall dissection: a contemporary overview for a proper decision-making approach

**DOI:** 10.3389/fcvm.2026.1774336

**Published:** 2026-04-07

**Authors:** Riccardo Scagliola, Giancarlo Passerone

**Affiliations:** 1 Cardiology Division, Cardinal G. Massaia Hospital, Asti, Italy; 2 Cardiothoracic Surgery Unit, University of Genoa, Genoa, Italy

**Keywords:** atrial wall dissection, decision-making approach, iatrogenic etiologies, imaging work-up, non-iatrogenic etiologies

## Abstract

Despite its clinical relevance and potentially life-threatening sequelae, atrial wall dissection remains an under-recognized and poorly understood condition in clinical practice. This review aims to provide a comprehensive and updated overview of atrial wall dissection focusing on the definition, etiopathogenesis, diagnostic work-up, and management of this uncommon entity. Regardless of the wide-ranging etiology, dissection sites, and clinical manifestations, physicians must be aware of such a rare finding to ensure a timely diagnosis and prompt identification of subjects with an unstable hemodynamic profile. This makes it possible to adopt the most appropriate decision-making strategy and therapeutic approach.

## 1 Introduction

Atrial wall dissection is an under-recognized and poorly understood entity in clinical practice. It typically develops as a complication of other conditions, either related to interventional procedures or as a result of non-iatrogenic findings. Unlike aortic dissection, atrial wall dissection is not primarily related to an intrinsic wall weakness and is generally caused by an injury. Time of presentation and clinical manifestations of atrial dissection are extremely diverse, ranging from incidental detection in asymptomatic subjects to acute onset with potentially life-threatening complications resulting in low cardiac output syndrome and hemodynamic collapse (1). Today's understanding of this rare event is still mainly based on sporadic case reports and case series described in the literature. This review aims to provide a comprehensive and updated overview of the etiopathogenesis, clinical findings, imaging diagnostic work-up, and therapeutic approaches of atrial wall dissection, with the goal to improve understanding of such a rare condition and raise awareness of its potential implications in clinical practice.

## 2 Methods

An extensive search of the literature on PubMed and Google Scholar was performed from January 1984 to December 2025. We searched for the following keywords (in Title and/or Abstract): (“atrial” OR “atrium”) AND (“dissection”). All the available English-language studies, registries, and case reports/series, with both abstract and full-text available, providing data about epidemiology, etiopathogenesis, clinical presentation, imaging work-up and management of atrial dissections were included in the research material. Of the 489 articles initially retrieved, 93 duplicates and 154 records in languages other than English were removed. Following through screening of literature search, 125 records were also ruled out as they not pertained to the research topic. Among the remaining 117 records, 53 were furtherly ruled out because of the following exclusion items: papers without full-text available; original articles; conference proceedings; editorials; letters to the Editor; and author's reply. Records were also excluded because of lacking individual case data or with insufficient data regarding clinical findings, imaging work-up and/or management of atrial dissection. Overall, 5 registries and 59 case reports/series were identified for final inclusion in our research material.

## 3 Definition, epidemiology and historical background

**Figure 1:**
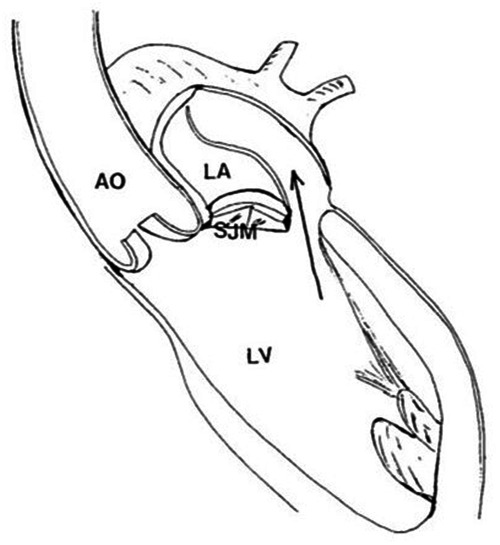
Schematic drawing depicting a dissection of the posterior left atrial wall, with the creation of a false cavity (arrow) compressing the true atrial chamber. The entry point of the dissection is localized in the posterior portion of the mitral prosthetic annulus [reproduced from Idir et al. (3). Ao, aorta; LA, left atrium; LV, left ventricle; SJM, St. Jude Medical prosthesis.

Atrial dissection is commonly defined as a contained separation of the atrial wall layers due to aberrantly pressurized blood flow. This creates a false blood- or thrombus-filled chamber between the atrial endocardium and myocardium, with or without communication with the true atrial chamber (1, 2) (Figure 1). It was firstly reported by Maguire et al. in 1887 in a patient with subaortic endocardial perforation and subsequent development of atrial septal dissection communicating with the left ventricular chamber (4). The detection of atrial dissections has significantly increased over the last 20 years, likely because of the continuous improvement in the imaging diagnostic accuracy and routine use of intraoperative transoesophageal (TOE) echocardiography (1). Most frequently atrial dissection involves the left atrium. It mainly occurs after surgical mitral procedures, with an incidence rate of 0.16% following mitral valve repair and 0.84% following mitral valve replacement (5, 6). Less than 5% of all reported cases were related to aortic valve surgery, while a smaller percentage was reported after isolated coronary artery bypass grafting (0.02%) or other cardiac interventions, for instance left ventriculoplasties or cardiac mass excisions (7–9). Recently, the number of reported iatrogenic atrial dissections has increased along with the number of patients undergoing catheter-based procedures over the past few decades (10, 11). Additionally, less than 20% of left atrial dissections have a non-iatrogenic etiology, mainly related to blunt chest traumas, systemic disorders, infective or degenerative valvular diseases (1, 12). Finally, right atrial dissections have also been anecdotally described, either following cardiac interventions or occurring in subjects with congenital heart diseases and/or right-sided structural abnormalities (13–15).

## 4 Etiopathogenesis

### 4.1 Iatrogenic causes

#### 4.1.1 Surgical etiologies

Several surgical causes of atrial wall dissection have been encountered. Surgical mitral valve repair or replacement with prostheses is the most common cause of iatrogenic atrial dissection. Extensive debridement of the posterior mitral annulus or subvalvular apparatus, inadequate suturing or traction applied to the annulus, inadvertent injuries to the atrial endocardium by surgical retraction, oversized prostheses, and inadequate reversal of anticoagulation may contribute to the occurrence of atrial dissection (16–19). Mitral annuloplasty may also predispose to atrial dissection, especially in case of sclerosis of floppy mitral valves, which are prone to ring dehiscence due to tissue friability (20). In accordance with the classification proposed by Treasure and coworkers, the interlayer dissection of the left atrium following mitral valve surgery can be categorized as an alternative form of type I left ventricular rupture, occurring at the level of the posterior atrioventricular groove (21, 22). Any primary endocardial tear may present a vulnerable entry point to pressurized blood flow, which can originate from the left atrial endocardium as a result of interventional tissue handling or surgical dissection, or from the left ventricular chamber through a paravalvular defect (1, 5). In rare cases, atrial dissection may develop following aortic valve replacement. In this surgical scenario, the division of the non-coronary aortic annulus in the region of the aortomitral continuity allows an easy opening of the aorto-atrial space, which is composed of loosely arranged muscle tissue bundles and can propagate the dissection into the atrial wall. Left atrial dissection has also been seldom reported after coronary artery bypass graft surgery (7, 23, 24). Pressurized inflow into the entry tear of the dissection originating from retrograde cardioplegia infusion is likely to play a pathogenetic role in this patient population. Specifically, cardioplegia supplement with a retrograde approach through the cannulation of the coronary sinus (CS) is a widely established pharmacological therapy during cardiac surgery. However, pressurized inflow through a displaced CS catheter, or forceful catheter insertion or repositioning carry a high risk of puncturing or perforating the CS wall, potentially leading to the development of atrioventricular groove hematoma and its propagation into the left atrial wall through the left atrioventricular sulcus (1, 7, 25). Additional surgical causes of atrial dissection have rarely been described, these include pulmonary vein cannulation, left ventricular venting tube misplacement, atrial mass excisions, or left ventriculoplasties (9, 17, 26–29).

#### 4.1.2 Catheter-based etiologies

Atrial dissection has also been reported following transcatheter procedures. Dissections related to percutaneous coronary interventions following distal wire perforation have seldom been noticed, accounting for approximately 0.3%–0.6% of total cases. Specifically, coronary perforations often occur following complex revascularizations (e.g., of chronic total coronary occlusions) and mainly involve the circumflex or distal right coronary arteries, because of their close proximity to the posterior left atrial wall (2, 10, 30). Furthermore, atrial dissection may also develop following structural heart procedures or transcatheter ablation of cardiac arrhythmias, either in relation to mechanical force from stiff wire manipulation, or to direct endocardial damage caused by radiofrequency energy application (11, 13, 31–33).

### 4.2 Non-iatrogenic causes

#### 4.2.1 Traumatic injuries

Blunt chest trauma is the commonest reported cause of non-iatrogenic atrial dissection. It refers to the heart bursting due to compression between the sternum and vertebral column, with an estimated incidence between 0.5% and 5%. Several causes may be responsible for blunt thoracic injuries, including high-speed impacts during motor vehicle collisions, sport injuries, blast forces, and iatrogenic procedures like cardiopulmonary resuscitation (12, 34). In the latter case, the application of excessive transmural pressure to the atrial walls during mechanical extrinsic compression generates high fluid shear forces. These may cause an entry tear along the atrial endocardium, and a distinct pocket-like cavity between the endocardial and myocardial layers (35–37). Similarly, traumatic injuries related to valvular regurgitation may predispose to developing atrial dissection and/or hematoma, following damage to the endocardial layer caused by the regurgitant jet and the resulting substantial systolic flow into the false lumen (38, 39).

#### 4.2.2 Infective endocarditis and mitral annular calcification

Infective endocarditis may predispose to non-iatrogenic atrial dissection, as it can lead to paravalvular abscess formation either following prosthetic valve interventions or necrosis of heavily calcified annular areas. This can progress into mycotic aneurisms, which may dissect the atrial wall and lead to fistulous communications between the cardiac chambers (16, 40). However, in specific circumstances, the presence of mitral annular calcification itself may predispose to the disruption of the left atrial endocardium creating an entry point for the dissection, especially when associated with advanced age, cardiovascular risk predictors, or chronic kidney disease (41).

#### 4.2.3 Mechanical complications of myocardial infarction

Tardive mechanical sequelae of myocardial infarction are anecdotally noticed as causative factors of atrial dissection, especially in patients with large infarcts or who do not receive timely coronary revascularization. Guerreiro and coworkers reported a case of left atrial free wall dissection complicating a left ventricular basal inferior pseudoaneurysm, which expanded through the mitral ring into the left atrium (42). Similarly, Kirsch and colleagues described a post-infarct rupture of the membranous ventricular septum, associated with the development of interatrial septal dissection (43).

#### 4.2.4 Congenital or acquired atrial wall weakness

Finally, atrial wall dissection may also develop from significant tissue friability and vascular frailty. This has been observed in connective tissue disorders or infiltrative diseases like cardiac amyloidosis, or in patients under chronic immunosuppressive treatment (12, 44). Additionally, atrial dissection may also be related to a congenital weakness of the atrial tissue, despite sporadic cases reported in the literature (15, 45).

## 5 Location

Different topographic sites of dissections within the atrial wall have been reported based on their surrounding etiologies (Table 1). Specifically, the posterior left atrial wall is the most likely site for developing atrial dissections, accounting for more than 80% of total cases (1). Unlike the anterior leaflet, the attachment of the posterior leaflet to the mitral annulus is primarily muscular, with little or no fibrous tissue. Furthermore, the posterior mitral leaflet and hemi-annulus are more prone to calcification as compared to the anterior leaflet, whose annular attachment is tougher and has fewer calcifications. These anatomical differences are postulated to make the posterior left atrial wall prone to dissections, especially following mitral valve surgery (2, 46, 47). In contrast, dissections affecting patients undergoing aortic valve surgery tend to occur along the interatrial septum and/or anterior atrial wall. This is because the disruption of the non-coronary annulus and the dissection propagation along the aortomitral continuity makes these atrial sites prone to layer separation in this population subset (7, 24, 48, 49). Dissections involving the interatrial septum are also seldom reported following transeptal puncture during catheter-based procedures, with an incidence reported at 0.5% of total cases. However, this is often underestimated, especially when considering transeptal approaches performed without intra-operative echocardiographic assistance, since the puncture site is not directly visualized (11, 50). In these cases, atrial septal dissections and/or hematoma may develop when the needle is oriented to the muscular atrial septum or the limbus of the fossa ovalis, or following the inadvertent puncture of other structures (32, 51). In case of uncommon resistance of the transeptal catheter, dissection of the interatrial septum should be suspected, and can be confirmed by septal staining with an iodine contrast medium (52). Other possible locations for the development of endocardial tears include the left atrial dome and/or pulmonary veins junctions, especially for dissections developing after pulmonary vein cannulation or electrophysiological procedures (8). The atrioventricular groove is considered another liable topographic region for developing atrial dissections, and a potential critical point for left ventricular rupture. This is because it provides a naturally weakened transitional area vulnerable to tears following overdistension, intense traction and forceful manipulation of the mitral annulus and subvalvular apparatus (46, 47). Moreover, its close proximity to the CS and circumflex artery makes the atrioventricular groove a critical site for the occurrence of left atrial dissection, particularly following cardiac surgery and percutaneous interventional procedures (1, 29).

**Table 1:** Characterizing features of atrial dissections.

Atrial dissection	Characteristics
Etiologies	Iatrogenic (>80%) • *Cardiac surgery* (Cardiac valve replacement/repair; coronary artery bypass graft surgery, surgical heart manipulation, cardiac mass excision, left ventriculoplasty) • *Catheter-based procedures* (Percutaneous coronary interventions, transcatheter ablations of cardiac arrhythmias, structural heart procedures) Non-iatrogenic (<20%) (Traumatic injuries, mechanical sequelae of myocardial infarction, infective endocarditis, degenerative valvular diseases, congenital or acquired wall weakness)
Location	• Posterior left atrial wall (>80%) • Interatrial septum/anterior left atrial wall • Other (left atrial dome and/or pulmonary veins junctions; atrioventricular groove; right atrial free wall)
Clinical findings	Extremely heterogeneous, ranging from asymptomatic subjects, up to potentially life-threatening complications resulting in low cardiac output syndrome and hemodynamic collapse
Diagnostic tools	Transoesophageal echocardiography is the imaging modality of choice for confirming atrial dissections and providing differential diagnosis
Prognosis	• Favourable outcomes (Absence of false chamber rupture; normal atrioventricular valve function; absence of interatrial shunting; contained septal or atrial free wall hematoma) • Worse outcomes (Signs of hemodynamic instability; impaired atrioventricular valve function; compression of the true atrial chamber; interatrial shunting; extended dissection hematoma beyond septal or atrial free wall layers)
Management	• Interventional repair (adopted for patients with signs of hemodynamic impairment) • Conservative approach (suggested for hemodynamic stable patients, with favourable clinical outcomes and echocardiographic findings)

## 6 Clinical findings and time of presentation

Atrial dissections may present as a wide range of nonspecific clinical manifestations, including chest pain, dyspnoea, palpitations, fatigue, asthenia and syncopal episodes. Chest pain is generally related to the development of an intimal tear as the entry point of pressurized inflow. Worsening dyspnoea and physical signs of congestive heart failure—including pulmonary crackles, ankle and/or abdominal swelling, newly detected S3 sound and/or jugular turgor—are generally the result of a progressive expansion of the false atrial chamber encroaching into the adjacent structures. This leads to a progressive obstruction of the left ventricular inflow and/or pulmonary veins by intramural hematoma (53). Furthermore, acute obstruction of mitral or tricuspid inflow may manifest as pulmonary oedema and/or cardiogenic shock, as a consequence of low cardiac output syndrome. This may lead to hypoxia and peripheral tissue damage due to poor systemic perfusion (46, 54, 55). Left atrial dissection may also predispose to developing cerebral ischemic events following the development of thrombus in the false atrial chamber due to blood stasis (18). Because of this, clinicians should consider cerebral embolism in patients with a diagnosis of left atrial dissection presenting with new-onset neurologic deficits. Additionally, potential arrhythmic complications like new-onset atrial fibrillation, are also possible, especially when the dissection chamber involves and/or encroaches on the pulmonary veins. Atrial dissection may also present as a chronic process incidentally discovered by routine follow-up imaging examinations in totally asymptomatic subjects. It may also show progressive hemodynamic deterioration due to a variety of cofactors, including gradually increasing of perivalvular leakage, the development of a local or systemic septic condition an increasingly fibrous and friable atrial wall (56, 57). Time of presentation and clinical significance of atrial dissections is widely variable, ranging from immediately intraoperative up to several years after the original precipitating injury. Most often they occur shortly after the causative agent, while a delayed or even late clinical presentation is less common. The time of clinical manifestation of atrial dissection can thus be classified by time patterns as: (i) “early”, occurring shortly after the causative procedure (hours or days); (ii) “delayed”, occurring within months after the precipitating injury; and (iii) “late”, occurring one or more years after the causative factor (1, 8).

## 7 Imaging tools and differential diagnosis

**Figure 2:**
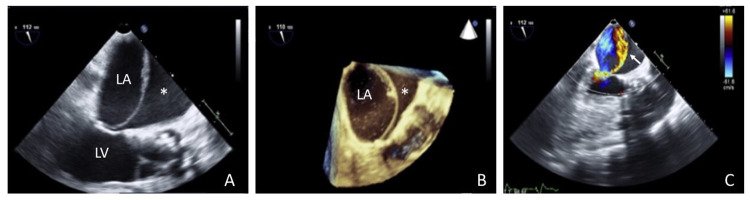
**(A,B)** Bidimensional and 3D visualization of left atrial dissection at transoesophageal echocardiography, with systolic expansion of the false lumen (asterisk). **(C)** Color Doppler showing a significant retrograde mitral regurgitation jet into the dissection chamber (white arrow) (adapted from Sardar et al. (31). LA, left atrium; LV, left ventricle.

Imaging assessment of suspected atrial dissection plays a pivotal role in providing the correct diagnosis, avoiding misleading interpretations, and informing further interventional treatments. TOE has been demonstrated to be mandatory in order to confirm an atrial dissection diagnosis. In this scenario, the echocardiographic appearance is typically dynamic, evolving from a hypoechoic non-obstructive space along the atrial wall to an expanding hyperechoic hematoma with potential inflow obstruction (Figures 2A–C) (31, 58, 59). Standard mid-oesophageal views are generally sufficient to provide a timely diagnosis; however, nonstandard off-axis views are often useful, especially for defining the potential retrograde extension of atrial compression to the afferent venous districts (29). The usual echocardiographic appearance of a suspected atrial dissection includes the presence of an echolucent gap extending from the mitral or tricuspid annular planes to the interatrial septum or atrial free wall. This results in a false cavity with or without communication with the true atrium, which may cause partial systolic obliteration of the true atrial chamber. Other possible echocardiographic findings include: periprosthetic mitral and/or aortic valve leaks (for dissections with iatrogenic valvular etiologies); new mitral or tricuspid regurgitation or left-to-right shunts detected using color Doppler analysis; signs of ventricular inflow stenosis; and caval or pulmonary venous flow obstruction (60–63). In certain situations, TOE examination may also provide a direct visualization of the entry point of the dissection, and/or the communication with the true atrial chamber. The atrial dissection flap has a homogeneous acoustic intensity and moves randomly; by contrast, linear artifacts show a gradually fading acoustic intensity, move with the cardiac motion, and extend beyond the anatomical atrial contours (60). However, the dissection entry point may not always be apparent, and detection is often challenging (18). In this specific scenario, color Doppler may help to examine the atrial endocardium and locate the flow source into the dissection, which is essential to guide surgical repair. When imaged, it shows a high-flow regurgitant jet, whose direction helps to identify the path of the propagating flow. Furthermore, color Doppler is useful for determining whether adjacent structures are compressed by showing any turbulent flow or fistulous communication (3, 17). Finally, color Doppler interrogation of the mitral and tricuspid valves, as well as each of the caval and/or pulmonary veins, demonstrating laminar flow, is essential to rule out venous flow obstruction (26). On the other hand, M-mode echocardiography may help in differentiating between true and false atrial chambers and distinguishing subtle motions of the atrial endocardial layer in relation to the cardiac cycle (60). Pulsed wave Doppler can be used for measuring blood flow speed across the tear and allows to distinguish high-pressure (ventricular) vs. low-pressure (venous) blood flow source. Additionally, it is helpful for assessing the venous flow pattern—especially when a venous flow obstruction by the dissection chamber and/or hematoma is suspected—or pathologic communication with the dissection cavity. A normal flow pattern rules out venous obstruction. The venous flow pattern shows a systolic blunting in case of atrial hypertension, while a diastolic blunting or a sudden cut in diastolic flow is suggestive of ventricular inlet obstruction (6, 26). The differential diagnosis of an expanding atrial mass may include several entities like atrial thrombi, tumours, cysts, vegetations, and coronary aneurysms (18, 29, 58). Other entities that should be considered when an atrial mass is detected include anatomical atrial variants such as septal pouches and/or ridges, atrial septal aneurisms, lipomatous atrial septal hypertrophy, embryonic remnants like Chiari network, doubled interatrial septum, and cor triatriatum (64, 65). All of these findings should be routinely investigated or ruled out by a preliminary initial intra-operative TOE examination with real-time imaging guidance for cardiac interventional procedures (e.g., for assisting in CS cannulation, structural heart interventions, and confirming the proper engagement of the fossa ovalis by the transeptal catheter) reducing the likelihood of injuries predisposing to iatrogenic dissections (17, 62). Intraluminal thrombi are the most commonly detected atrial masses, and may resemble the intermediate stage of atrial dissections. However, they usually originate in the left atrial appendage, have a typically mobile or free-floating echogenicity, and are unlikely in fully anticoagulated patients. Other differential diagnoses include the presence of extensive pericardial hematomas, which may cause an extrinsic compression of the atrial chambers similar to that caused by a thrombosed atrial dissection. However, in the latter case, its initial echocardiographic appearance is of a filamentous structure originating from the atrial endocardial surface, which temporally evolves into an intramural echogenicity (7). Therefore, atrial dissection should be suspected in patients with unexplained hemodynamic impairment and the presence of specific echocardiographic insights. This is especially true in subjects undergoing cardiac interventions or with other predisposing conditions, once potential diagnostic pitfalls have been ruled out. Under specific circumstances, the echocardiographic findings of atrial dissection are quire confusing and may resemble conditions causing extrinsic cardiac compression like cysts, extracardiac masses, and loculated cardiac tamponade, especially when the dissection is focal and contains clots. Consequently, while TOE is the imaging technique of choice for diagnosing atrial dissections, when echocardiographic findings are unclear, a multimodality imaging approach including the use of cardiac computed tomography (CT) and/or magnetic resonance imaging (MRI) may be helpful determining the best diagnostic work-up and decision-making approach (18, 66–68) (Table 2). Specifically, cardiac CT provides an accurate anatomical evaluation of the dissection site due to its high spatial resolution, although its temporal resolution is currently lower than both CMR and TOE. Furthermore, cardiac CT can be a reliable tool for a better understanding of cardiac anatomy, in order to plan and guide interventional treatment via minimally invasive or standard approaches (40, 67). On the other hand, cardiac MRI provides more accurate information on tissue characterization, and is able to confirm the anatomical continuity between the atrial dissection and the surrounding atrial wall. Additionally, by using different pulse sequences, it helps detect any haemorrhagic component in the lesion (compatible with an involving parietal hematoma) as well as the concurrent presence of atrial thrombus. As a result, in hemodynamically stable patients cardiac MRI can be a useful tool to confirm the healing of the dissection, and closely monitor the dissection site in the follow-up period (2, 68, 69). However, in some cases, atrial wall dissection can be challenging to diagnose even when several imaging tools are applied, Therefore, in specific cases only surgical exploration can clarify the nature of the atrial lesion (18).

**Table 2:** Comparative imaging findings for diagnosis of atrial dissection.

Imaging tool	Advantages	Disadvantages
Transoesophageal echocardiography	• Widely availability • Early evaluation of critical ill patients • Greater accuracy in assessing valvular anatomy and function • Immediate feedback on atrial wall dysfunction	• Semi-invasive technique • Often requiring sedation • Lack of myocardial tissue characterization • Potential risk of mechanical complications • Not suitable for all patients
Computed tomography	• High spatial resolution • Fast scan times • Multiplanar reconstruction capability	• Low temporal resolution • Radiation exposure • Less ideal for detailed functional assessment of cardiac valves and atrial chambers
Magnetic resonance imaging	• High temporal resolution • Detailed tissue characterization • Potential assessment of heart and valve function. • No radiation exposure	• Slow scan times • Lower spatial resolution compared to computed tomography • Not suitable for patients with certain metal devices

## 8 Therapeutic approaches and recommendations

**Figure 3:**
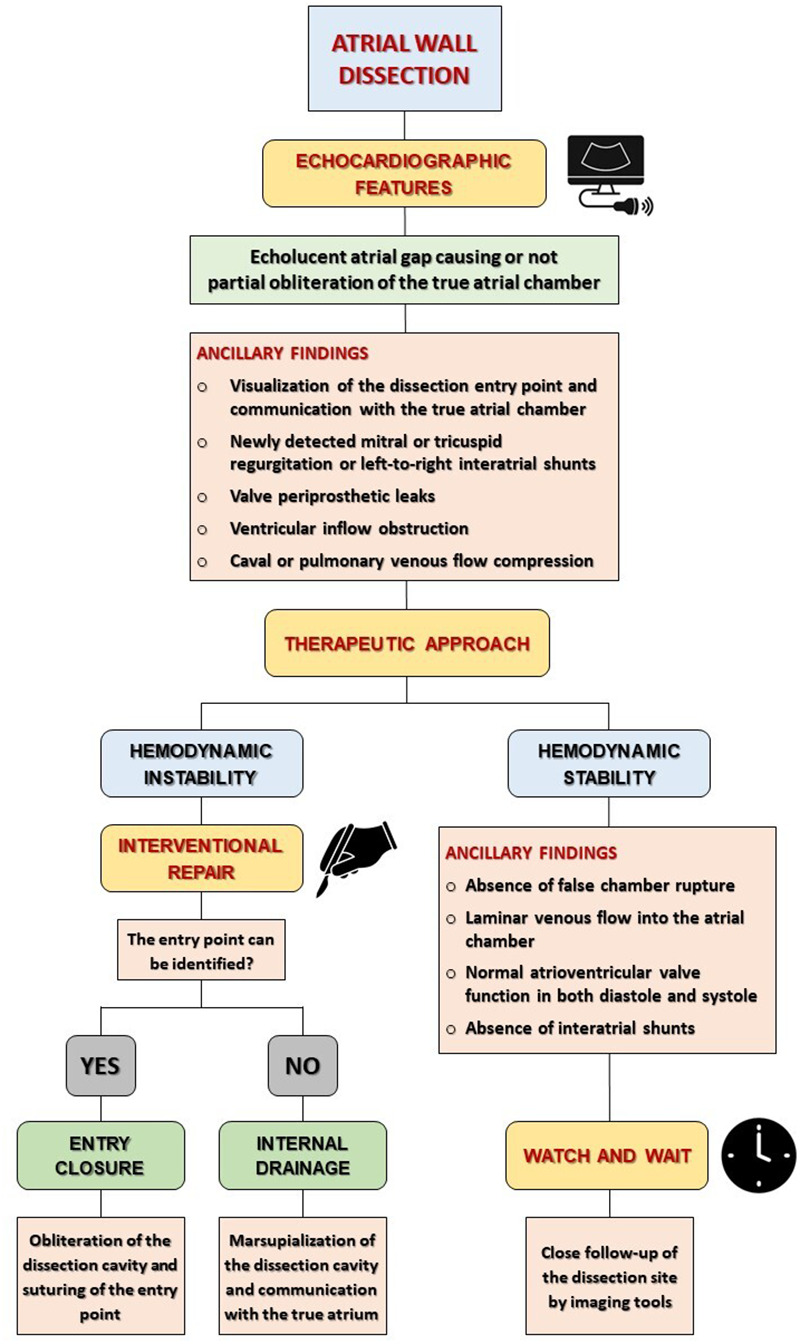
Proposed flow-chart for diagnosis and decision-making approach of atrial dissections.

To date, the optimal management of atrial dissection is still under debate. Despite the lack of definitive criteria and guidelines for the management of atrial dissections, a number of clinical aspects are key in planning a proper therapeutic work-up. In case of signs of hemodynamic impairment indicative of a rapid expansion of the atrial dissection hematoma and compression of the true atrial chamber, a prompt surgical approach is highly recommended (3, 9, 38). Two different surgical strategies are encountered: entry closure and internal drainage. Most surgical approaches focus on the evacuation of the hematoma from the dissection cavity, obliteration of the false atrial chamber, and closure of the dissection entry injury by edge-to-edge mattresses suture or using a bovine pericardial or synthetic patch (8, 12, 55). Internal drainage of the dissection cavity is preferred when the entry point of the dissection cannot be identified and consists of the marsupialization of the false cavity, which is allowed to communicate with the true atrial chamber. However, this technique is more prone to recurrences, as the open entry point can lead to a persistent pressurized inflow (7, 70). There are only few reports of percutaneous treatment of atrial dissection describing an alternative therapeutic strategy especially in critical patients with a prohibitive surgical risk (71). Conversely, a “watch and wait” approach with regular follow-ups is suggested for hemodynamically stable patients with favourable clinical and prognostic outcomes (56, 58). Additional echocardiographic findings supporting a conservative approach include: the absence of false chamber rupture; laminar venous blood flow into the atrial chamber; normal atrioventricular valve function in both diastole and systole; and absence of interatrial shunting (65) (Figure 3). As reported by Fukuhara and colleagues, less than 25% of atrial dissection subjects were managed conservatively, with the majority of them undergoing surgical treatment (1). In patients with iatrogenic atrial septal dissections, the decision should be individualized, according to the hemodynamic presentation and the extent of the lesion. For subjects with contained septal hematoma, the outcome is usually favourable and a surgical procedure associated with continued anticoagulation therapy is likely a safe option. Conversely, special attention should be paid to patients with a hematoma extending beyond the interatrial septum, as the progression of the hematoma into the atrial free wall may compress the true atrial cavity and induce hemodynamic collapse (48, 52). Through surgical correction and conservative treatment are both viable options in selected cases, to date not enough long-term follow-up investigations have been carried out. Therefore, the therapeutic approach for patients with atrial dissection should be individually tailored based on the patient's clinical and haemodynamic profile, in order to maximize the benefits and minimize the risk of inappropriate surgical interventions. Further investigation is advisable in order to provide clearly defined diagnostic criteria and shared guidelines for a consistent decision-making approach.

## 9 Conclusions

Albeit uncommon and often under-recognized, atrial dissection remains a critical finding in clinical practice. Regardless of its cause, the topographic heterogeneity of atrial sites, and wide range of clinical manifestations, physicians must be aware of such a rare condition, its potential implications and diagnostic pitfalls and clues. This ensures a timely diagnosis and prompt identification of patients with a critical hemodynamic profile in order to adopt the most appropriate decision-making strategy and therapeutic approach. Further investigation is still needed to fill in the gaps in the current understanding of such uncommon finding in clinical practice.
